# Tissue Distribution Study of Poloxamer188 in Rats by Ultra-High-Performance Liquid Chromatography Quadrupole Time of Flight/Mass Spectrometry with MS^ALL^-Based Approach

**DOI:** 10.3390/molecules26185644

**Published:** 2021-09-17

**Authors:** Yixuan Feng, Lele Li, Yuxuan Li, Xinxin Zhou, Xiaoying Lin, Yue Cui, Heyun Zhu, Bo Feng

**Affiliations:** 1 School of Pharmacy, Jilin Medical University, Jilin 132013, China; fengyixuan77@163.com (Y.F.); lilele19911006@163.com (L.L.); liyuxuan1995426@163.com (Y.L.); z13843214189@163.com (X.Z.); linxytime@163.com (X.L.); 2 School of Life Sciences, Jilin University, Changchun 130012, China

**Keywords:** poloxamer188, UHPLC-Q-TOF/MS, tissue distribution, quantitative analysis

## Abstract

Poloxamer188 (PL188), as one of the most commonly used pharmaceutical excipients, has unique physicochemical properties and good biocompatibility, and so is playing an increasingly extensive role in the field of medicine. Currently, there are few studies on the tissue distribution of PL188 in vivo. In this study, the LC-MS method based on MS^ALL^ technique of quadrupole time of flight mass spectrometry for absolute quantitative analysis of poloxamer 188 in biological substrates was established for the first time. The tissue distribution of poloxamer188 in SD rats were studied using the established quantitative analysis method. To explore the distribution of PL188 in organs and tissues, PL188 was administered via rat tail vein at a dose of 5 mg/kg. Eight kinds of tissues including heart, liver, spleen, lung, kidney, stomach, muscle and brain of rats were collected at 0.25 h, 1 h and 4 h after administration. Tissue distributions showed the highest level was observed in kidney, then in stomach, which indicated PL188 mainly bioaccumulated in the kidney. This study can provide references for the further study of PL188.

## 1. Introduction

Poloxamers, also known as Pluronics, are synthetic nonionic copolymers with an A-B-A type triblock structure comprising a central hydrophobic chain of poly propylene oxide (PPO) (B) flanked by two hydrophilic chains of poly ethylene oxide (PEO) (A) [1]. Poloxamers exhibit an amphiphilic character in aqueous solution on the basis of the PEO solubility and the PPO insolubility. The PEO blocks are hydrophilic, while the PPO block is hydrophobic [2]. Based on the alternating distribution of polar and nonpolar segments, poloxamers can form unimeric micelles or higher-ordered aggregates in aqueous solution, which demonstrably feature surface-active properties. The size and structure of poloxamer assemblies, and their adsorption properties, have made them useful in many applications, including: drug delivery [3,4], nanoparticle synthesis [5], emulsion formulation [6] and ophthalmic pharmaceutical formulations [7], to name a few. The use of poloxamers, more specifically, Poloxamer188 (PL188), in pharmaceutical research is widely researched [8,9,10]. However, to date, there are a few reports describing the tissue distribution of PL188 in vivo [11,12]. Accordingly, the aim of this study was to develop an accurate and robust analytical method to simultaneously determine PL188 in biological samples and apply it to the tissue distribution study.

Currently, a number of analytical techniques have been applied to the determination of Poloxamers including colorimetric methods [13], various chromatographic techniques [14,15], etc. However, many of them suffer from disadvantages such as poor selectivity and reproducibility, low sensitivity, long analytical run time, requirement of a large sample volume, and complex sample preparation. Multiple reaction monitoring (MRM) based on liquid chromatography mass spectrometry (LC-MS) offers sufficient sensitivity and selectivity to simultaneously determine analytes in biological samples [16,17]. However, it fails to scan many ion pairs and, in many cases, the use of MRM of certain precursor→product ion transitions is not applicable for the determination of PL188 in vivo. Recently, a novel method based on MS^ALL^ technique of UHPLC-Q-TOF/MS plate for the determination of polymers was reported [18,19]. Quadrupole time of flight/mass spectrometry (Q-TOF/MS) is a new hybrid mass spectrometry consisting of a unit mass resolution quadrupole (q1), a second quadrupole (q2) and a TOF mass analyzer with high mass resolution. It has two basic MS/MS scan modes, namely product ion scan and MS^ALL^ scan. The principle of product ion scan is similar to MRM in LC-MS/MS. It only quantitates a limited number of precursor ions. In contrast, MS^ALL^ is a scan mode specific to Q-TOF/MS which is generally used for qualitative analysis in metabolomics [20,21] and proteomics [22,23]. It can perform data-independent fragmentation of all ions entering the mass spectrometer in one scan, and acquire both high resolution MS and MS/MS data using low and high collision energy (CE) in different experiments for a single injection of sample. Since all precursor ions of PL188 can be fragmented regardless of their *m*/*z*, MS^ALL^ has the potential to be a fully comprehensive technique for the analysis of polymer drugs.

In this study, we developed a novel and robust MS^ALL^ method based on Q-TOF/MS in positive ion mode. All precursor ions of poloxamer formed in the ion source are sent to the collision cell (q2) for fragmentation, and all product ion information of poloxamer can be obtained. Data so acquired can then be mined by using narrow window extracted ion chromatograms. PL188 was selected as a model to study the tissue distribution of poloxamers in rat after intravenous injection. Finally, the results demonstrate that MS^ALL^ analysis is a strategy with accurate and robust for the quantitation of PL188 in organs and tissues of rats.

## 2. Results and Discussions

### 2.1. Optimization of MS Conditions 

**Figure 1:**
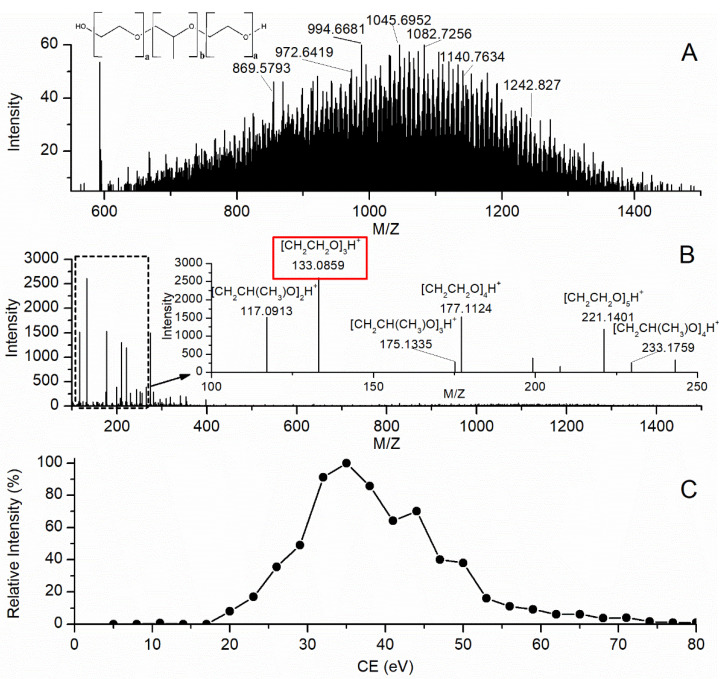
Full scan spectrum and chemical structure of PL188 (**A**), MS/MS spectrum of PL188 based on MS^ALL^ mode (**B**), Signal intensity of product ion (*m*/*z* 133.0859) of PL188 with respect to CE based on MS^ALL^ mode (**C**).

Following infusion into the Q-TOF MS from a syringe pump, PL188 standard solutions were full scanned in TOF-MS mode with CE set at the minimum value (5 eV) and declustering potential (DP)adjusted at 100 eV. Figure 1A illustrated full scan analysis of PL188. The compound produced complex precursor ion spectra in the range of *m*/*z* 500–1500 with no single ion peak resolved. Because poloxamers are polydisperse, PL188 molecules contain polymers with a wide range of molecular weights. As a result, MRM of precursor→product ion transitions are not universally applicable to its analysis in biological samples.

Figure 1B shows MS^ALL^ analysis for PL188 at the high CE value of 35 eV. In this case, generated spectra of charged ions with the singly charged ions produced from PL188 differed in mass by 44 or 58 Da, thus allowing the number of ethylene oxide or propylene oxide subunits in each ion to be determined from its *m*/*z* value (product ions with *m*/*z* 133.0869, 177.1124 and 221.1401 contain sequentially 3 to 5 ethylene oxide subunits, product ions with *m*/*z* 117.0913, 175.1335 and 233.1759 contain sequentially two to four propylene oxide subunits), as the structure of PL188 in Figure 1C shows. The specific ion of PL188 at *m*/*z* 133.086 gave a higher intensity than any of the PL188 related ions. Therefore, it was chosen for quantitation. CE values which ranged from 5 to 80 eV were further optimized to get the maximum intensity of the quantitative ion (*m*/*z* 133.0859). As shown in Figure 1C, the intensity of the product ion at *m*/*z* 133.0859 maximized at CE of 35 eV.

**Figure 2:**
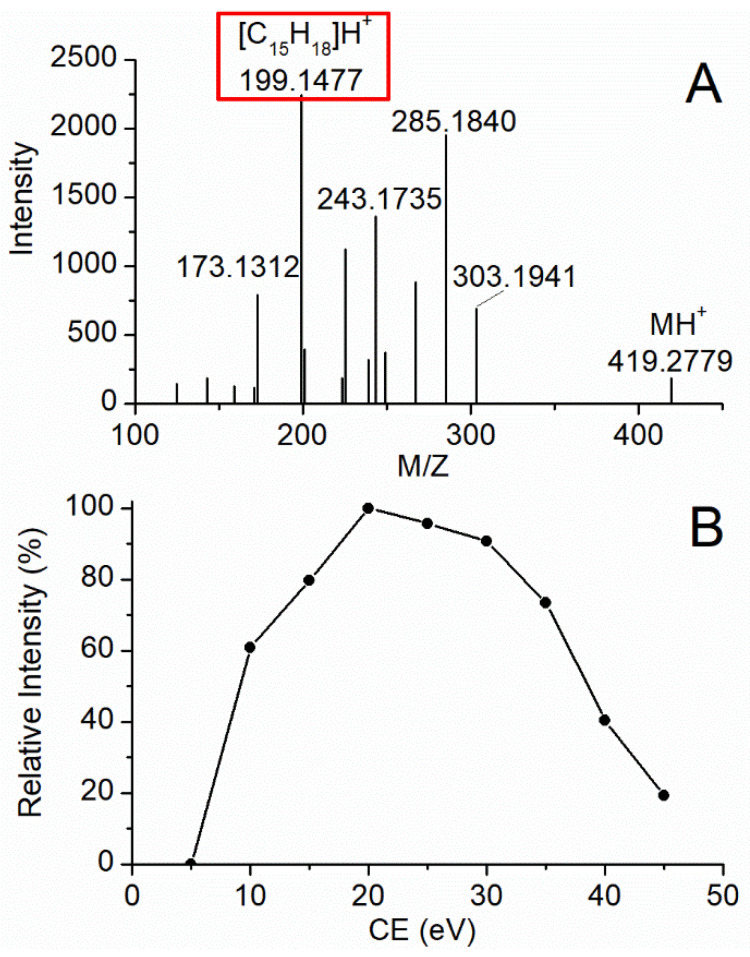
MS/MS spectrum of simvastatin (IS) (**A**) and signal intensity of product ion (*m*/*z* 199.1477) for IS with respect to the CE based on product ion mode (**B**).

Product ions of simvastatin (IS) were scanned in product ion mode and Figure 2A illustrated the product ion analysis for IS at CE of 20 eV. As shown in Figure 2A, *m*/*z* 199.1477 gave the best signal-to-noise ratio and was therefore chosen for quantitation. CE values, which ranged from 5 to 55 eV, were further optimized to get the maximum intensity of the ions at *m*/*z* 199.1477. As shown in Figure 2B, the intensity of the product ion at *m*/*z* 199.1477 maximized at CE of 20 eV.

### 2.2. Chromatography Development

PL188 with a series of large molecular weights have strong retention on reversed phase columns, so the pore size of the solid particles of the stationary phase should also be considered in selecting chromatography column. For the separation of large molecules such as proteins, peptides and PL188, the particles must have large pores (microporous, ≥300 Å) in order to ensure good interaction with analytes and reduce the tendency of large molecules to block the pores. A gradient elution with 0.1% formic acid-acetonitrile: isopropanol (40:60, *v*/*v*) and 0.1% formic acid in water was used as mobile phases. The PLRP-S reversed-phase column (1000 Å, 4.6 × 50 mm, 8 μm) which has large pores and provides excellent stability at 1–14 pH, was used to separate PL188 in complex biological matrix.

### 2.3. Assay Validation

Assay validation includes specificity, linear range, LLOQ, accuracy, precision, dilution effect, recovery, carryover, crosstalk, matrix effects and stability.

**Figure 3:**
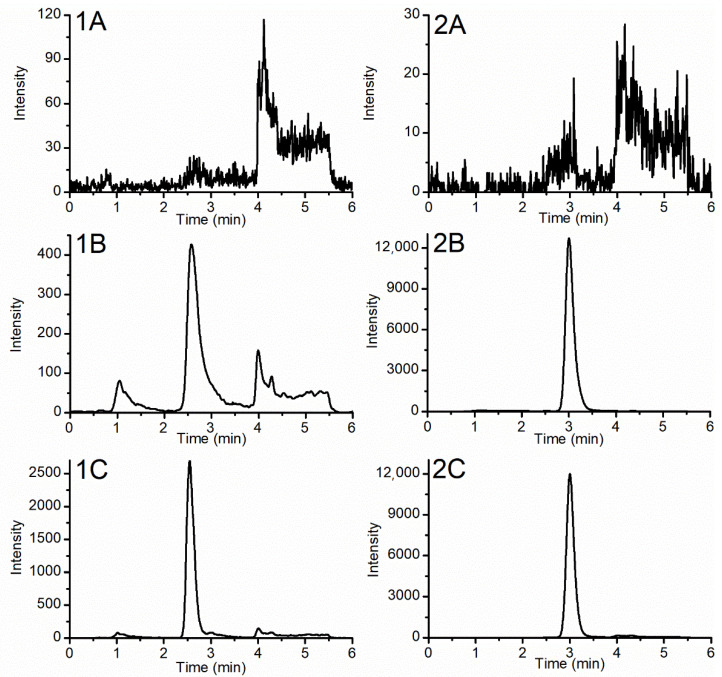
Representative extracted ion chromatograms of blank liver (**A**), blank liver sample spiked with PL188 and IS (**B**) and liver sample after administration of PL188 (**C**). (1) PL188 and (2) IS.

The extracted ion chromatograms of PL188 (product ion 133.0859) and IS (product ion 199.1477) are shown in Figure 3. There was no significant peak at the retention time of PL188 and IS indicated the assay was free of interference which showed a good specificity. Testing of linearity showed the assay was linear over the range of 0.1–10.0 μg/mL with LOD of 0.03 μg/mL for PL188. The typical regression equation was y = 0.1964x + 0.04020 (*r* = 0.9976). Intra- and interday accuracy and precision were all within accepted limits (±15%) at QC and LLOQ concentrations as shown in Table 1. Assay of samples with high concentration after a 10-fold dilution also gave satisfactory results with calculated concentrations in the range 85–115% of the nominal concentrations. Extraction recoveries of PL188 at low, medium and high concentrations were 85.7 ± 6.1%, 75.2 ± 9.2% and 73.9 ± 3.8%, respectively (As shown in Table 2). Corresponding matrix effects of nominal concentrations were 93.0 ± 5.4%, 88.6 ± 7.3% and 89.2 ± 6.5%, respectively (As shown in Table 2). These results indicated that ion suppression and enhancement were not significant for PL188. In terms of carryover, sequential analysis of ULOQ and blank plasma samples showed no obvious peak in the latter. In addition, crosstalk between PL188 and IS was shown to be absent. As shown in Table 3, The RSDs and REs of preprocessed stability, postprocessed stability, autosampler stability, freeze-thaw stability and long-term stability were less than 13%. Stability evaluations showed that PL188 were stable under all the evaluated conditions.

### 2.4. Tissue Distribution

**Figure 4:**
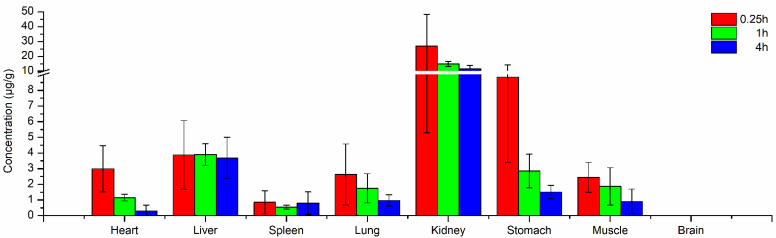
Mean contents of PL188 in different tissues (μg/g) at 0.25, 1 and 4 h after an intravenous administration of 5.0 mg/kg PL188 to rats. (Mean ± SD, *n* = 4).

The changing trends of PL188 concentrations in different organs and tissues at different time points of 0.25, 1, and 4 h after administration were investigated. Figure 4 reveals that PL188 distributed rapidly and widely in different organs and tissues excepting brain. PL188 passed through the blood vessel wall firstly. The pore size of the blood vessel wall is different in various organs and tissues, so the content and speed of drug distribution to each tissue are also different. Generally, drugs with small molecular weight can pass the blood vessel wall rapidly. The drug then entered the extracellular fluid and finally penetrated the cell membrane of the tissue.

The highest mean concentration of PL188 was found in the kidney (26.80 μg/g at 0.25 h), which was significantly higher than that in other organs. The result illustrated that PL188 could pass through the blood vessel wall of the kidney easily, and the blood flow ratio of kidney is high which also benefited the distribution of PL188 in the kidney. The concentration of PL188 in kidney was 11.63 μg/g at 4 h, which was about half of that at 0.25 h. Therefore, the elimination rate of PL188 was low in kidney. The high concentrations in kidney might be related to the bioaccumulation of PL188 in this tissue, which would lead to potential organ toxicity after repeated administration.

The higher concentrations of PL188 in stomach and liver were also related to the higher blood flow ratio of these two tissues. There are abundant blood vessels in portal veins and the gastrointestinal system. Therefore, the high concentrations of PL188 in the stomach might be related to the bioaccumulation of PL188 in this tissue. Besides, the concentrations of PL188 in liver were always high from 0.25 h to 4 h, This might related to the large molecular weight of PL188 which made it is decomposed slowly in liver. 

The concentrations of PL188 in lung, muscle, and spleen were low. The concentrations of poloxamer188 lung decreased from 2.63 μg/g at 0.25 h to 0.97 μg/g at 4 h, and the concentrations of poloxamer188 in muscle decreased from 2.45 μg/g at 0.25 h to 0.91 μg/g at 4 h. These results indicated that there was no bioaccumulation of PL188 in the two tissues. No PL188 was detected in rat brain which indicated that PL188 could not enter into the brain through the blood-brain barrier.

## 3. Materials and Methords

### 3.1. Chemical Reagents and Materials

Poloxamer188 was purchased from MREDA (Beijing, China). Simvastatin used as internal standard (IS) was purchased from Sigma-Aldrich (St. Louis, MO, USA). Ultra-high-purity water was prepared using a Milli-Q System. Methanol, isopropanol, acetonitrile and formic acid of LC-MS grade were purchased from Thermo Fisher (San Jose, CA, USA).

Eighteen male Sprague-Dawley rats (180–220 g) were purchased from the Experimental Animal Center of Jilin University (Jilin, China). The experimental protocol was approved by the Animal Ethics Committee of Jilin Medical University (Approval Code: 20210135, Approval Date: March 15th, 2021), and all animal studies were carried out in accordance with the Guidelines for Animal Experimentation.

### 3.2. Sample Preparations

The various organs and tissues (heart, liver, spleen, lung, kidney, stomach, muscle and brain) were harvested and rinsed with ice-cold 0.9% NaCl to remove the superficial blood. After being blotted dry with filter paper, each weighed tissue sample was homogenized using tissue homogenizer in physiological saline solution (1:4, *w*/*v*). Then, samples were centrifuged at 1600× *g* for 5 min. The supernatant was taken and processed using the following processing method.

PL188 solution (1.0 mg/mL) was prepared in acetonitrile: water (40:60, *v*/*v*). Calibration standards were prepared by diluting stock solutions with blank tissue homogenate of liver to the final concentration of 0.1, 0.2, 0.5, 1.0, 2.0, 5.0 and 10.0 μg/mL. Lower limits of quantitation (LLOQ, 0.1 μg/mL) and QC samples (0.3, 1.5 and 8.0 μg/mL) were prepared independently in the similar method. Simvastatin (internal standard, IS) was dissolved in methanol and diluted with acetonitrile: water (40:60, *v*/*v*) to give a 1 μg/mL IS working solution.

Calibration standards, LLOQ, QC and tissues samples for analysis (50 μL) were mixed with 50 μL IS working solution and 200 μL cold 0.1% formic acid-acetonitrile: water (40:60, *v*/*v*), vortexed for 30 s and centrifuged at 7500× *g* for 5 min. The supernatant was injected into LC-MS for analysis.

### 3.3. UPLC-Q-TOF/MS Conditions

The chromatographic separation was performed on a LC-20AD Prominence^TM^ ultra-high-performance liquid chromatography (UHPLC) system (Shimadzu, Japan) coupled with PLRP-S Reversed-Phase column (1000 Å, 4.6 × 50 mm, 8 μm; Agilent Technologies, Palo Alto, CA, USA) maintained at 40 °C. Mobile phase A was 0.1% formic acid in the water, and mobile phase B was 0.1% formic acid-acetonitrile: isopropanol (40:60, *v*/*v*). The proportion of mobile phase B was used as follows: 40% (0–1.4 min), 40–65% (1.4–1.5 min), 65–65% (1.5–3.0 min), 65–95% (3.0–3.1 min), 95–95% (3.1–4.5 min), and then back to 40% at 4.6 min for 1.4 min of equilibration. The injection volume was set to 40 μL, and the flow rate was set as 0.8 mL/min.

Q-TOF/MS analysis was carried out on a Triple-TOF 5600+ MS (SCIEX, Concord, Canada) equipped with an ESI source. MS parameters were optimized by direct infusion of standard solutions via a syringe pump as follows: positive ion mode; source temperature 600 °C; ion spray voltage 5500 V; nebulizer gas (N_2_) 55 psi; heater gas (N_2_) 60 psi; curtain gas (N_2_) 35 psi; declustering potential 100 V. PL188 was scanned in TOF-MS scan mode with collision energy 35 eV, and MS^ALL^ data were acquired from *m*/*z* 100 to 1500. IS was scanned in product ion mode using *m*/*z* 419.2 for parent ion and a range of *m*/*z* 100–450 for product ion, the collision energy value of IS was 20 eV.

### 3.4. Data Processing

Data acquisition was controlled by Analyst 1.7.1 software (SCIEX, Concord, ON, Canada). Both peaks of PL188 and simvastatin were integrated using specific integration parameters in Multiquant 3.0.2 (SCIEX, Concord, ON, Canada). The quantitative product ion of PL188 and simvastatin were *m*/*z* 133.0859 (from 133.0809 to 133.0909) of MS^ALL^ data and *m*/*z* 199.1477 (from 199.1422 to 199.1522) of product ion data, respectively. Linear least-squares regression of calibration curves with 1/x^2^ weighting were used to evaluate linearity.

### 3.5. Assay Validation

This method was fully validated in accordance with the US-FDA document and other related guidelines with respect to specificity, linearity, precision and accuracy, recovery, matrix effect and stability [24].

Six blank liver homogenate, liver homogenate spiked with PL188 and IS and liver samples after administration were used to evaluate the specificity. Linearity was evaluated by linear least-squares regression with a weighting index of 1/x^2^ of calibration curves based on the peak area ratios of PL188 and IS, and calibration curves were constructed for each batch. Intra- and interday accuracy (evaluated by relative error, RE) and intra- and interday precision (evaluated by relative standard deviation, RSD) were based on the assay of six replicate QC samples on three different days. LLOQ was defined as the lowest concentration that could be determined with accuracy and precision ±20%. The extraction recovery of PL188 was determined at three QC levels and calculated by comparing the analyte standard peak areas obtained from postextracted samples with extracted samples. Matrix effects were evaluated by comparing peak areas of PL188 and IS in postextraction spiked samples with those in standard solutions. Carryover was calculated by analyzing extracted blank rat plasma samples after analysis of a sample at the upper limit of quantitation (ULOQ). Crosstalk interference between analyte and IS was evaluated by analyzing an ULOQ sample followed immediately by blank rat plasma spiked with IS working solution. Stability was evaluated using three QC levels. The QC samples were stored under different environments: at room temperature for 4 h before the process, at room temperature for 4 h after the process, in an autosampler at 4 °C for 8 h after the process and freeze-thaw cycles from −20 °C (freeze) to room temperature (thaw) three times, and storing at −80 °C for 30 days (long-term stability). 

### 3.6. Tissue Distribution

Male Sprague-Dawley rats, weighing 200 ± 20 g, were obtained from the Experimental Animal Center of Jilin University (Changchun, China). The rats were divided into three groups (four rats for each time point). After the rats were administrated of PL188 (5 mg/kg) through caudal vein, tissue specimens (heart, liver, spleen, lung, kidney, stomach, muscle, brain) were collected at 0.25, 1 and 4 h post-dosing, respectively. The tissue harvesting and homogenizing method were given in Section 2.

## 4. Conclusions

This study described the development, validation and application of a method using Q-TOF/MS based on MS^ALL^ technique for the comprehensive and quantitative analysis of PL188 and its application to the tissue distribution of PL188 in rats. The results showed that the PL188 distributed most in kidney, followed by liver and stomach. The high concentrations might be related to the bioaccumulation of PL188 in these tissues which might lead to potential organ toxicity. Besides, PL188 was not accumulated in lung, muscle, and spleen, and could not enter into the brain through the blood-brain barrier. It is anticipated that this method will be widely applied in the study of poloxamers in excipients, emulsifiers, and wetting agents’ fields.

## Figures and Tables

**Table 1:** Accuracy and precision of PL 188 in rat liver (*n* = 6).

Concentration (μg/mL)	Intraday		Interday	
	Accuracy (RE, %)	Precision (RSD, %)	Accuracy (RE, %)	Precision (RSD, %)
0.1	9.7	6.8	12.5	10.9
0.3	8.3	5.4	7.6	12.1
1.5	−1.1	7.2	2.2	9.3
8	−6.9	8.1	−8.4	6.7

**Table 2:** Matrix effect and extraction recovery results of PL188 in rat liver (*n* = 6).

Concentration (μg/mL)	Matrix Effect		Extraction Recovery	
	Mean ± SD (%)	RSD (%)	Mean ± SD (%)	RSD (%)
0.3	93.0 ± 5.4	5.8	85.7 ± 6.1	7.1
1.5	88.6 ± 7.3	8.2	75.2 ± 9.2	12.2
8	89.2 ± 6.5	7.3	73.9 ± 3.8	5.1

**Table 3:** Stability results of PL 188 in rat liver (*n* = 3).

Concentration (μg/mL)	Before Process (4 h, RT)	After Process (4 h, RT)	Auto-Sampler (8 h, 15 °C)	Three Freeze-Thaw Cycles (−20 °C to RT)	Long-Term (−80 °C, 30 days)
	RE (%)	RE (%)	RE (%)	RE (%)	RE (%)
0.3	−3.6	1.2	2.8	−4.4	−6.7
1.5	−3.1	−8.5	−5	−6.2	−9.6
8	−10.2	−7.7	−11.3	−10.9	−13

## Data Availability

The data presented in this study are available on request from the corresponding author.

## Sample Availability

Samples of the compounds are available from the authors.

## Abbreviations

PL188	poloxamer188
PPO	poly propylene oxide
PEO	poly ethylene oxide
MRM	multiple reaction monitoring
Q-TOF/MS	Quadrupole time of flight mass spectrometer
CE	collision energy
DP	declustering potential
IS	internal standard
LLOQ	lower limit of quantitation
QC	quality control
LOD	limit of detection
ULOQ	upper limit of quantitation
UHPLC	ultra-high-performance liquid chromatography
